# Family-Centered Social Cognitive Factors Preventing Primary Tooth Caries in Children Based on Intervention Mapping Approach

**DOI:** 10.1155/2021/6626090

**Published:** 2021-05-18

**Authors:** Bahareh Kabiri, Ali Reza Hidarnia, Mehdi Mirzaei Alavijeh, Mohammad Esmaeel Motlagh, Ali Montazeri

**Affiliations:** ^1^Health Education and Health Promotion, Faculty of Medical Sciences, Tarbiat Modares University, Tehran, Iran; ^2^Professor, Department of Health Education and Health Promotion, Faculty of Medical Sciences, Tarbiat Modares University, Tehran, Iran; ^3^Department of Health Education and Health Promotion, School of Health, Kermanshah University of Medical Sciences, Kermanshah, Iran; ^4^School of Medicine, Jundishapur University of Medical Sciences, Ahvaz, Iran; ^5^Population Health Research Group, Health Metrics Research Center, Iranian Institute for Health Sciences Research, ACECR, Tehran, Iran

## Abstract

**Background:**

Given the increasing prevalence of primary tooth caries in Iran and the importance of providing evidence- and theory-based family-centered prevention programs, the present study is aimed at determining the family-centered social cognitive factors preventing deciduous tooth caries among children using the intervention mapping protocol.

**Methods:**

This cross-sectional study was performed on 240 Iranian mothers in Ilam who were randomly selected to participate in the study. The data were collected using a self-designed questionnaire including items on demographic information and social cognitive constructs (knowledge, attitude, perceived severity, perceived benefits, perceived barriers, practice guidance, perceived self-efficacy, behavior intention, subjective norms, and social norms). The questionnaire was completed by mothers, and the data were analyzed by performing one-way analysis of variance and linear regression.

**Results:**

The results obtained from linear regression analysis showed that perceived self-efficacy (*B* = 0.295, *p* < 0.001), perceived barriers (*B* = 0.084, *p* < 0.028), practice guidance (*B* = 0.774, *p* < 0.001), and social norms (*B* = 0.137, *p* < 0.020) accounted for 71% of the behavioral intention variance and were the most important predictors for preventing primary tooth caries among children.

**Conclusion:**

The findings suggest that perceived self-efficacy, perceived barriers, practice guidance, and social norms are essential for developing family-centered programs to prevent primary tooth caries in children.

## 1. Introduction

Primary teeth start to grow about 6 months after birth and complete their growth when children are 3-5 years of age so that they can satisfy their nutritional needs [1, 2].

Primary teeth form the foundation of the permanent ones and are very susceptible to caries. Therefore, maintaining their health is considered a serious matter for children's health because caries in primary teeth is a major risk factor for its occurrence in permanent teeth [3, 4].

Despite being a very preventable infectious disease, dental caries is still one of the most common chronic childhood diseases, strongly influences the health and social and intellectual development of communities by causing problems such as pain, difficulty with eating and speech impairments, and imposes exorbitant costs on families and communities [5–7]. Globally, early childhood caries (ECC) prevalence reported in the world varies from 60 to 90 percent with the developed countries having the lowest and the developing countries the intermediate and/or highest prevalence [8]. The American Academy of Pediatrics (AAP) emphasizes that specialist healthcare for identifying effective factors in oral and dental health in all children must begin at the age of 6 months. Therefore, the first year of life is considered the “critical period” for general or oral and dental health [9, 10].

Parents are the first among the social forces that influence children's growth and health in their early years of life. They also play an important role in the development of children's healthy habits, continuation of their healthy behaviors, and prevention of diseases [7].

Mothers play a vital role in children's growth and development, spend a considerable amount of time with them, are directly responsible for their oral and dental health, and play an important role in preventing oral and dental diseases in them [11, 12]. Since oral and dental diseases have a relatively high prevalence in Iran and as no studies based on families and mothers have been conducted in this relation in this country, it is necessary to use a framework for determining predictors related to behaviors that prevent dental caries in primary teeth. There are various available models and theories for health education, but among them, the capability of intervention mapping in planning programs for health promotion has been confirmed in different studies. The intervention mapping protocol addresses issues with the problem-solving perspective using the ecological approach. It was first introduced in 1998 by Kay Bartholomew and Professor Guy Parcel from the University of Texas in Houston in the USA and Professor Kok from Maastricht University in Holland in the Journal Health Education & Behavior. This protocol has 6 steps: Step 1—the Needs assessment; Step 2—the purposes in changing the behavior of the individuals and environmental factors; Step 3—the selection of theory-based intervention methods and selection or preparation of practical strategies; Step 4—the planning of the intervention program; Step 5—the planning of the programs or their adoption, implementation, and sustainability; and Step 6—the evaluation plan. This research intended to determine the cognitive and social factors related to prevention of dental caries in children by their mothers using the two steps of needs assessment and the objectives in changing the behavior of individuals and environmental factors in the intervention mapping protocol [13, 14].

## 2. Materials and Methods

This cross-sectional study was performed on mothers of children aged 6 months to 1 year in Ilam, Iran. The mothers were selected through the random stratified sampling method.

A total of 240 mothers were selected using the following formula:

(1) $$\begin{array}{c} n=\frac{\sigma^{2}*\;z_{1-\left(\alpha /2\right)}^{2}}{d^{2}},\\ n=\frac{{\left(0.5\right)}^{2}*{\left(1.96\right)}^{2}}{{\left(0.2\right)}^{2}}240. \end{array}$$

Samples were selected from mothers visiting all 18 health centers in Ilam in proportion to the population of patients at each health center. The inclusion criteria were having a 6-month- to 1-year-old child, having a healthy child, having a health record, and literacy, and the exclusion criteria were pregnancy, mother and child underlying disease, separation, and divorce (because the presence of these factors in the mother may affect the process of child care by the mother).

Data were collected using a self-designed questionnaire. The structure of this questionnaire was designed within the intervention mapping approach [15–20]. The questionnaire's items were then discussed and reviewed by the research team. The questionnaire contained items on demographic information such as child's age (month), mother's age (year), mother's education (illiterate/primary, secondary, and higher), mother's occupation (housewife, unemployed, and employed), spouse's age (year), spouse's education, and spouse's occupation (retired, unemployed, and employed) and items on social cognitive constructs including 13 items for assessing the knowledge (Cronbach's *α* = 0.75) with score ranging from 0 to 26, for example, “primary teeth caries may result in permanent teeth caries,” 5 items for assessing the attitude (Cronbach's *α* = 0.71) with score ranging from 5 to 25, for example, “It is important for me that my child have healthy and beautiful teeth,” 4 items in the perceived severity domain (Cronbach's *α* = 0.81) with score ranging from 4 to 20, for example, “Deciduous teeth caries may result in permanent teeth caries,” 4 items in the perceived benefits domain (Cronbach's *α* = 0.71) with score ranging from 4 to 20, for example, “If my child has healthy primary teeth, he will have healthy permanent tooth,” 4 items in the perceived barriers domain (Cronbach's *α* = 0.76) with score ranging from 4 to 20, for example, “Busy life does not allow me to brush my child's teeth every night,” 5 items in the perceived self-efficacy domain (Cronbach's *α* = 0.78) with score ranging from 5 to 25, for example, “I can provide healthy snacks for my child,” 5 items in the behavior intention domain (Cronbach's *α* = 0.70) with score ranging from 5 to 25, for example, “I decided to brush my child's teeth after each meal,” 3 items in the practice guidance domain (Cronbach's *α* = 0.73) with score ranging from 3 to 15, for example, “My spouse reminds me not to forget my child's brush,” 4 items in the social norms domain (Cronbach's *α* = 0.74) with score ranging from 4 to 20, for example, “It is important for me what others think and say about my child's oral mouth,” and 4 items in the subjective norms domain (Cronbach's *α* = 0.76) with score ranging from 4 to 20, for example, “My friends concern about their child's oral health.” It should be noted that all items were scored on a Likert scale from 1 (strongly disagree) to 5 (strongly agree). The instrument validity was determined through content validity ratio and content validity index. To assess the content validity index, the questionnaire was provided to 10 health education and health promotion professionals and pediatric dentists, and the necessary corrections were made) all of them were experts in the field of oral health and had done a lot of research in the field of oral health.(

In addition, the construct validity was determined through the exploratory factor analysis. The data were analyzed by performing linear regression, one-way analysis of variance using the SPSS-21 software.

## 3. Results

All 240 mothers participated in the study. The mean (SD) age of mothers was 31.85 (5.67) years. Moreover, 56.3% of mothers had a university degree, and 72.9% were housewives.  Table 1 shows the demographic characteristics of the participants.

According to the correlation test, there was a statistically significant relationship between maternal age with knowledge, perceived severity, perceived benefits, and perceived self-efficacy. In addition, there was a significant relationship between the child age and two constructs of behavior intention and practice guidance of mothers, so that with increasing age, the behavior intention of mothers to take care of their children's oral care increased.  Table 2 shows this relationship.

Linear regression analysis was performed to examine the variables affecting oral health behaviors among children. As presented in Table 3, Model 6 explained 71% of the variance for oral health behaviors among children. This model was obtained from combining perceived barriers, perceived self-efficacy, practice guidance, and social norms constructs. In addition, there was no significant difference between preventive behaviors for deciduous teeth decay and underlying variables.

## 4. Discussion

Oral health has to be definitely considered an important part of the human organism and not an isolated segment [21].

The findings showed that the perceived self-efficacy, perceived barriers, practice guidance, and social norms were four major and effective cognitive determinants in preventing deciduous tooth caries in Iranian children. The impact of perceived self-efficacy is a reflection of mothers' perceptions of the ease or difficulty of the intended behavior. According to Bandura, self-efficacy is the strongest predictor of behavioral change in the individuals [22]. Since one of the most powerful tools to increase self-efficacy is having adequate skills in performing a behavior, thus, changes in self-efficacy may occur following successful and active participation of individuals in the desired behavior. Many studies have confirmed the role of perceived self-efficacy as an influential construct on behavior [23, 24]. In the field of oral health, several studies have shown that perceived self-efficacy was one of the strongest determinants of behavior [25, 26]. In a study, Anagnostopoulos et al. reported that perceived self-efficacy and perceived severity were the predictors of the brushing behavior frequency [27]. Similarly studies by Padula and Sullivan and Pimsurang et al. showed that perceived self-efficacy and perceived barriers were two strong predictors of oral health behaviors [28, 29]. However, some studies have emphasized on the role of objective perceived severity as a powerful predictor of oral health behaviors [30]. Other studies have emphasized on the role of attitude, positive social outcomes, and perceived behavior control in the field of oral care. To achieve oral health behaviors, these studies have suggested ways to develop a more positive attitude toward social norms in individuals [31, 32].

Some studies have emphasized on the role of anxiety and concern as important factors that have a negative impact on oral health and have stated that positive social consequences are effective in having good and healthy teeth [33, 34].

In studies by Johns, Champion, and Stretcher, perceived barriers were the strongest predictor of behavior, and reducing them was one of the best programs to influence oral self-care [35].

The current showed that mothers who received more support from people such as dentists, family members, and especially spouses had more intention for oral health care of their children. Many other studies on different health behaviors have also shown the role of support and positive external incentives, so that being reminded by relatives, the impact of abstract norms and the effect of influencers on mothers' behavior were important determinants of oral health care [36, 37].

A study showed that 65% of the changes in brushing behavior were related to the extended theory of planned behavior [38]. In other studies, the attitude, subjective norms, and perceived behavior control predicted 66.2% and 63% of the behavior intention variance in the context of oral health; meanwhile, perceived behavior control was more effective than other variables and was the most powerful predictor of behavior intention [25, 39]. However, we did not find a significant relationship between underlying variables and social cognitive constructs. In some studies, parental education was the most important determinant of children's socioeconomic status, and there was a more inverse relationship between parental education and caries in developed and developing countries [16]. There are other findings that are consistent with the present study and do not support such a relationship [40, 41]. Nevertheless, family has an essential role in public health and oral health and is a source for acceptance and transmission of health information, such that health behaviors are constructed and established within the family [42]. Therefore, the role of parents should be taken into consideration when planning for oral health behaviors. In addition, educational programs on children's oral health should reinforce the idea that, despite perceived barriers, parents can observe oral health of their children through promoting self-efficacy. In addition, any planning to reduce barriers will help promote health behaviors.

## 5. Conclusion

The findings showed that cognitive factors, especially four determinants of perceived self-efficacy, perceived barriers, practice guidance, and social norms, have an effective role in preventing primary tooth caries in children. Therefore, it seems necessary to consider the cognitive components when designing intervention programs for preventive behaviors.

## Figures and Tables

**Table 1:** Demographic characteristics of the study subjects.

	Number (*n* = 240)	Percentage
*Child gender*		
Male	128	53.3
Female	112	46.7
*Mother education*	240	100
Secondary	105	43.7
Higher	135	56.3
*Father education*	240	100
Primary	27	11.2
Secondary	78	32.5
Higher	135	56.3
*Mother occupation*	240	100
Housewife	175	72.9
Employed	46	19.2
Unemployed	19	7.9
*Father occupation*	240	100
Employed	232	96.7
Unemployed	5	1.2
Retired	3	1.3

**Table 2:** The linear regression model for the constructs of knowledge, attitude, severity, benefits, barriers, self-efficacy, practice guidance, behavior intention, subjective norms, and social norms.

	*B*	SEB	Standardized B	*T*	*p* value
*Step 1*					
Knowledge	0.018	0.043	0.022	0.426	0.671
Attitude	0.098	0.069	0.078	1.434	0.153
Severity	0.061	0.052	0.059	1.178	0.240
Benefits	0.035	0.093	0.022	0.375	0.708
Barriers	0.082	0.039	0.101	2.085	0.038
Self-efficacy	0.234	0.066	0.230	3.538	0.001
Practice guidance	0.745	0.088	0.466	8.461	8.461
Social norms	0.103	0.068	0.091	1.512	0.132
Subjective norms	0.072	0.067	0.065	1.087	0.278
*Step 2*					
Knowledge	0.017	0.043	0.021	0.396	0.692
Attitude	0.091	0.066	0.072	1.386	0.167
Severity	0.059	0.051	0.058	1.150	0.251
Barriers	0.082	0.039	0.101	0.101	0.038
Self-efficacy	0.227	0.063	0.223	3.588	0.001
Practice guidance	0.743	0.088	0.465	8.472	0.001
Social norms	0.103	0.068	0.091	1.508	0.133
Subjective norms	0.071	0.066	0.064	1.069	0.286
*Step 3*					
Attitude	0.097	0.064	0.077	1.514	0.131
Severity	0.065	0.049	0.063	1.322	0.187
Barriers	0.082	0.039	0.101	2.093	0.037
Self-efficacy	0.232	0.062	0.228	3.768	0.001
Practice guidance	0.742	0.088	0.464	8.481	0.001
Subjective norms	0.103	0.068	0.091	1.510	0.132
Social norms	0.071	0.066	0.064	1.076	0.283
Step 4					
Attitude	0.091	0.064	0.072	1.425	0.155
Severity	0.068	0.049	0.066	1.382	0.168
Barriers	0.086	0.039	0.106	2.203	0.029
Self-efficacy	0.249	0.060	0.245	4.182	0.001
Practice guidance	0.744	0.088	0.465	8.505	0.001
Social norms	0.141	0.058	0.124	2.411	0.017
*Step 5*					
Attitude	0.102	0.064	0.081	1.604	0.110
Barriers	0.096	0.039	0.118	2.499	0.013
Self-efficacy	0.269	0.058	0.264	4.646	0.001
Practice guidance	0.752	0.088	0.470	8.593	0.001
Social norms	0.141	0.058	0.125	2.419	0.016
*Step 6* ^∗^					
Barriers	0.084	0.038	0.102	2.209	0.028
Self-efficacy	0.295	0.056	0.290	5.299	0.001
Practice guidance	0.774	0.087	0.484	8.925	0.001
Social norms	0.137	0.059	0.121	2.336	0.020

^∗^Final model: adjusted *R*‐squared = 71.131 and *p* < 0.001.

**Table 3:** Correlation coefficient matrix between cognitive and background variables.

	Mother age	Father age	Child age
Knowledge	0.18^∗∗^		
Perceived severity	0.23^∗∗^	0.16^∗^	
Perceived benefits	0.22^∗∗^	0.17^∗^	
Perceived self-efficiency	0.20^∗∗^		
Behavior intention			0.19^∗∗^
Practice guidance			0.12^∗^

^∗^Significant at 0.05 level. ^∗∗^Significant at 0.01 level.

## Data Availability

Data will be available if needed.
